# Effect of the COVID-19 outbreak and lockdown on mental health among post-secondary students in the Grand Est region of France: results of the PIMS-CoV19 study

**DOI:** 10.1186/s12955-021-01903-9

**Published:** 2021-12-15

**Authors:** Cédric Baumann, Hélène Rousseau, Cyril Tarquinio, Martine Batt, Pascale Tarquinio, Romain Lebreuilly, Christine Sorsana, Karine Legrand, Francis Guillemin, Stéphanie Bourion-Bédès

**Affiliations:** 1 EA4360 APEMAC, MICS Team, University of Lorraine, 54500 Vandoeuvre-lès-Nancy, France; 2 Méthodology, Data Management and Statistic Unit, University Hospital of Nancy, 54500 Vandoeuvre-lès-Nancy, France; 3 InterPsy, GRC Team, University of Lorraine, 54000 Nancy, France; 4 Clinical Investigation Center, CIC-EC, University Hospital of Nancy, 54500 Vandoeuvre-lès-Nancy, France; 5 Centre Psychothérapique de Nancy, 54520 Laxou, France

**Keywords:** COVID-19, French students, SF-12, Mental health, Living conditions

## Abstract

**Background:**

The COVID-19 epidemic has sent students around the world in to lockdown. This study sought to assess the prevalence of impaired self-perceived mental health and identify associated factors among French post-secondary students during the lockdown.

**Methods:**

A cross-sectional study was conducted among French students living in the Grand Est area in France from May 7 to 17, 2020 during the first lockdown. An online survey was used to collect sociodemographic data, learning and teaching conditions, living conditions, and exposure to COVID-19, and self-perceived mental health was assessed with mental composite score (MCS) of the SF-12.

**Results:**

Overall, 4018 were analyzed. Most participants were female (70.7%), and the mean age was 21.7 years (SD 4.0). The mean MCS score was 44.5 (SD 17.3). Impaired mental health, defined by a MCS < 1st Quartile, was mainly associated with female sex; decreased time for learning; not having access to the outside with a garden, a terrace or a balcony; difficulties with the living situation and having someone in the home affected by the SARS-COV2 requiring hospitalization or not.

**Conclusions:**

This study showed that living conditions during lockdown had a clear impact on the mental health of French post-secondary students. There is a need to improve prevention and to access distance education as well as an urgent need for measures to develop healthy coping strategies for students. This is significant challenge and will assist in moderating the risk for the development of further distress and mental health concerns.

## Introduction

The coronavirus disease 2019 (COVID-19), first identified in Wuhan in the east of China in December 2019 [1], has spread at an alarming rate and has become a major challenging public health problem around the world [2]. In response to this pandemic, the World Health Organization (WHO) declared a public health emergency of international concern on January 30, 2020 [3]. The mortality rate of COVID-19 was initially lower outside than in China [4], which led to the expectation that the disease’s impact on national health would be minor, which was not the case. In France, as in many countries, government officials announced the closure of middle and high schools, universities and other educational institutions to prevent the rapid spread of COVID-19 by breaking important chains of transmission [5]. This preventive measure concerned more than 2.7 million students.

Students have been identified as a vulnerable group experiencing significant levels of stress, anxiety and depression affecting their mental health [6, 7]. Before the pandemic, one in five college students worldwide experienced one or more diagnosable mental disorders [8, 9]. The academic years are considered an essential element in building a foundation of positive health behaviors that promote well-being into adulthood [10]. During this period of life, young adults face a variety of challenges and are exposed to significant risks affecting their health status [11]. A change in place of residence, increased responsibility, peer pressure, learning and work scheduling are all sources of psychological difficulties such as stress, depression and anxiety [12]. With years of budget cuts and the inability to meet basic student needs, these same students are even more vulnerable in such a crisis, and current evidence shows that students from lower sociodemographic backgrounds are more affected [13, 14].

Previous works showed that during an outbreak, individuals experience negative emotional responses, such as anxiety and depression symptoms [15]; hence, stressful events and public health emergencies such as the COVID-19 outbreak are potent adverse environmental factors that can have more psychological effects among students that can be expressed as fear, worry, altered quality of sleep and finally altered quality of life [16, 17].

Recent research among Chinese college students revealed that 24.9% experienced anxiety because of the COVID-19 outbreak. Overall, living in urban areas, living with parents and having a steady family income were protective factors against altered mental health for college students, whereas having a relative or acquaintance with COVID-19 was an independent negative risk factor [18]. Among French students, 25% experienced moderate to severe anxiety. Female sex and having relatives or acquaintances in the home who were hospitalized for COVID-19 were the main risk factors for anxiety [19].

Another problem during the lockdown concerned the potentially excessive use of the Internet. Indeed, in times of social isolation, the Internet is an obvious solution to maintain a social link with others. However, students are particularly fond of the Internet and may tend to overuse it [20]. Studies clearly show that excessive Internet use has a negative impact on students’ mental health [21].

Another study reported worsened health-related quality of life and decreased quality of sleep among Greek students during the lockdown [22]. Of course, self-perceived health status significantly varies according to the main socio-demographic characteristics of individuals. The level of general self-perceived health is increased among men, the youngest people, people living in a couple relationship or those with the highest incomes and qualifications. It also varies by employment status because unemployed people judge their health more negatively, particularly in the mental health dimension [23]. However, we do not know how this unprecedented health situation affects the mental dimension of quality of life of students according to the conditions of the lockdown.

With this recent literature on psychological and mental health impacts of the pandemic among students and because the Grand Est region was one of the three regions in France that was the most severely affected by the COVID-19 outbreak, the students of this area may have been at particular risk of severe mental health issues. Thus, to evaluate the self-perceived mental health of students and to understand their needs in order to develop appropriate responses, this study aimed to determine the socio-demographic and living conditions associated with impaired mental health among students in the Grand Est region during the first COVID-19 lockdown in France.

## Methods

### Design and sample

This study is a cross-sectional analysis of data from the PIMS-CoV19 study, an observational study involving an online survey from May 7 to 17, 2020, during the first lockdown in France. A sample of students was recruited from the University of Lorraine and the Sciences Po College located in Nancy, Lorraine, Grand Est region, France. The Grand Est Region ranked among the French regions the most affected by COVID-19 in terms of cases, with 19.6 cases per 100,000 inhabitants during the survey.

All students received detailed information regarding the purpose of the study and provided online informed consent to participate in the study. The survey was anonymous to ensure the confidentiality and reliability of data. All procedures were conducted in accordance with the principles of the Declaration of Helsinki and the study protocol was approved by the Institutional Review Board (Comité National de l’Informatique et des Libertés—registration 2,220,408).

### Data collection

The survey questionnaire consisted of three parts: sociodemographic data and living conditions during the lockdown and measurement of perceived mental health status assessed by a self-administered questionnaire. All data were collected at the time of the online survey.

#### Sociodemographic and other characteristics

Students self-reported their demographic characteristics, including age, sex, living arrangement, home location, and academic demographic information, including academic program and scholarship status. They were also asked about their living and learning conditions, changes in their consumption of psychoactive substances, preventive behaviors regarding COVID-19 and presence of a relative or acquaintance with COVID-19.

#### Mental health status

Students were asked to complete the Medical Outcome Study Short-form 12 (SF-12) questionnaire [24]. Two composite scores can be calculated: a physical component summary and a mental component summary (MCS). These scores range from 0 to 100 (100 = best perceived heath status). The SF-12 questionnaire was chosen for its good psychometric properties and for its relatively short completion time, recommended in this type of online survey. Only the MCS score was used in this study.

### Expected number of participants

When the study was launched, it targeted the entire student population in Lorraine (i.e., more than 50,000 students). We had estimated that 5% to 10% of the students would respond to the survey (from 2500 to 5000 students). No a priori sample size was calculated.

### Statistical analysis

Continuous variables are described with mean (SD) or median and categorical variables with proportions (percentages). To determine the model for the MSC, we first checked for deviations from normality and linearity of the MCS distribution. Thus, we analyzed the MCS in binary form. Because the objective of the study was to target students with highly impaired mental health and because of no recommended cut-off value for the MCS, we chose the first MSC quartile to split the sample (i.e., impaired vs not impaired mental health).

Logistic regression models were created to determine variables associated with the probability of impaired mental health status, an MCS score less than the first quartile. We investigated sociodemographic characteristics, learning and teaching conditions, the effect of living conditions and concerns regarding the threat to health posed by COVID-19. Relevant factors were associated on bivariable analysis at the 10% threshold. The suitability of the full model was evaluated and compared with a model with stepwise selection of candidate variables, using a significance entry level of 0.1 and significance staying level of 0.05. Odds ratios (ORs) and 95% confidence intervals (CIs) were estimated. The goodness of fit was assessed by calculating the model determination coefficient (R2) and the percentage that was predicted correct by the model. Pearson correlation, Phi coefficients and variation inflation factors (VIF < 10) were calculated to verify the lack of correlation and multicollinearity [25]. Hosmer and Lemeshow test allowed the comparison and selection of the best multivariable model. Analyses were performed with SAS 9.4 (SAS Institute Inc., Cary, NC, USA).

## Results

The target population was nearly 52,000 students. During the opening period of the online questionnaire, 9560 connections were recorded and 6021 students read the information note and agreed to participate. Overall, 5306 students completed at least one item of the questionnaire; 4018 completed the SF-12 questionnaire. No correlation was observed between the explanatory variables (all < 0.5). VIFs were consistently < 2, which indicated a lack of multicollinearity. The model determination coefficient (R2) was 0.19, and the percentage predicted correct was 73.2%.

### Sociodemographic and learning characteristics (Tables 1 and 2)

**Table 1:** Sociodemographic and living characteristics of post-secondary students in Grand Est region of France during the COVID-19 lockdown, May 7 to 17, 2020

	%
Characteristic	
Age mean (SD)	21.7 (4.0)
Gender	
Male	29.3
Female	70.7
Living arrangements	
Alone	13.9
With friends or a partner	19.8
With parents or family member	66.1
Financial aid scholarship	
None	59.5
Scholarship	40.5
Home location	
Urban area	59.4
Rural area	40.6
Access to an outside area	
No access	17.2
Private balcony, courtyard or terrace	15.5
Private domestic garden	60.1
Courtyard or garden for collective use	7.3
Difficulty isolating at home	
Yes	25.1
No	74.9
Tensions and conflicts at home	
Yes	28.3
No	71.7
Noises outside the home	
Yes	23.5
No	76.5
Noises inside the home	
Yes	19.8
No	80.2
Part-time job	
None	69.6
Activity interrupted during the lockdown	14.4
Activity increased during the lockdown	7.7
No change during the lockdown	8.3
Someone infected with SARS-COV2 at home	
No	84.4
Confirmed and hospitalized cases	0.7
Confirmed and non-hospitalized cases	3.7
Suspected cases	11.2
Relative or acquaintance infected with SARS-COV2	
No	49.6
Confirmed and hospitalized cases	12.0
Confirmed and non-hospitalized cases	22.4
Suspected cases	16.0

**Table 2:** Learning conditions and life styles of university students during the lockdown

	%
*Learning conditions*	
Academic program	
Sport, Medical sciences, Science and technology	57.9
Law, economics, management	17.3
Arts, humanities, languages	8.5
Social and human sciences	16.0
Online teaching delivery	
None	20.8
Partial online teaching	43.2
Total online teaching	36.0
Time working at home	
No change	29.2
Increased time working	20.1
Reduced time working	50.7
Postponement of final competition (Yes)	13.4
*Life styles*	
Frequency of exiting the home during lockdown	
Several times a day	4.2
Once a day	10.9
Several times a week	18.4
Once a week	19.0
Never or less than once a week	47.5
Alcohol consumption	
None	34.4
No change	16.8
Increased consumption	13.7
Reduced consumption	35.1
Tobacco consumption	
None	83.4
No change	3.0
Increased consumption	7.2
Reduced consumption	6.4

The sociodemographic and learning characteristics of the 4018 students are in Table 1. Most participants were female (70.7%) with mean age 21.7 years (SD 4.0). Participants were mostly from faculties of sciences, including sport sciences, science and technology and medical sciences (57.9%), followed by students in faculties of law, economy and management (17.3%) then social sciences (16%), and art, letters and languages (8.5%). Of the 4018 students, 40.5% reported financial aid and 14.4% faced an interruption of their student part-time job due to the lockdown. The time working at home decreased for 50.8% and 20.8% did not receive online teaching. With the lockdown, 13.4% reported a postponement of a final examination.

### Living conditions and behavior characteristics (Tables 1, 2 and 3)

**Table 3:** Means used by students to mitigate impaired mental health during the lockdown (N = 4018)

Option (%)	Not used	Ineffective 1	2	3	4	Very effective 5
Media entertainment	2.1	5.9	11.2	21.8	26.3	32.7
Reading entertainment	22.7	5.4	13.9	18.7	20.6	18.8
Physical exercise	16.9	5.1	10.7	16.6	20.3	30.3
Snacking between meals	24.1	11.7	19.7	18.8	13.6	12.1

Before the lockdown, one quarter of students resided in the parental home. During the lockdown, 13.8% lived alone, 19.8% with friends or a partner and 66.1% with their parents or a family member. More than half lived (59.4%) in urban areas, and 17.2% reported having no access to the outside such as a garden, terrace, or balcony. Of the 4018 participants, 28.3% reported conflicts with others where they lived. Before the lockdown, 17.5% reported consuming alcohol two or more times per week, and under the lockdown, 13.7% reported increased alcohol use. The means the most used to relieve stress were media entertainment (97.9%) and physical exercise (83.1%). One-third (34.4%) reported a relative or acquaintance infected with SARS-COV2, and 4.4% were living with someone infected with SARS-COV2 at home. Finally, nearly half of students (47.5%) reported never leaving home or less than once a week during the lockdown.

### Mental health status during the lockdown and associated factors

The mean MCS score was 44.5 (SD 17.3) (median 42.4, interquartile range 30.2–58.6), and 1004/4018 students had a score lower than 30.2 (the first quartile).

Table 4 shows the results of bivariable and multivariable analyses. We present the results of only multivariable analysis. Impaired mental health was associated with female sex (OR 1.5, 95% CI 1.3–9.9) and decreased time for learning (OR 1.6, 95% CI 1.3–1.9).

**Table 4:** Factors associated with impaired mental health status during the lockdown (N = 4018)

	Bivariate regression analysis			Multivariate logistic regression analysis R^2^ = 0.19 – H&L = 0.1		
	OR	95% CI	*P* value	OR	95% CI	*P* value
Gender (female vs male)	2.0	1.7–2.3	< 0.001	**1.5**	1.3–1.9	< 0.001
Age	01.0	0.9–1.0	< 0.001			
Home location (ref: urban vs rural area)	1.2	1.0–1.3	0.06			
Financial aid scholarship (ref: scholarship vs none)	1.1	1.0–1.3	0.13			
Access to a private outside area from home			< 0.001			< 0.001
Private domestic garden	1			1		
Private balcony, courtyard or terrace	1.2	0.9–1.4		1.2	0.96–1.5	
Courtyard or garden for collective use	1.1	0.9–1.5		1.3	0.9–1.8	
No access	1.5	1.3–1.9		**1.8**	1.4–2.2	
Difficulty isolating at home (Yes vs No)	2.3	2.0–2.7	< 0.001	**1.4**	1.2–1.7	0.0005
Tensions and conflicts at home (Yes vs No)	2.8	2.4–3.2	< 0.001	**2.1**	1.8–2.6	< 0.001
Noises outside the home (Yes vs No)	1.9	1.6–2.2	< 0.001	**1.3**	1.1–1.6	0.002
Noises inside the home (Yes vs No)	2.6	2.2–3.0	< 0.001	**1.5**	1.2–1.8	< 0.001
Someone infected with SARS-COV2 at home (ref: no)			< 0.001			0.005
Confirmed and hospitalized cases	3.4	1.6–7.4		**3.1**	1.3–7.2	
Confirmed and non-hospitalized cases	1.5	1.1–2.2		1.6	1.1–2.3	
Suspected cases	1.4	1.1–1.7		1.2	0.9–1.5	
Relative or acquaintance infected with SARS-COV19 (ref: no)			< 0.001			
Confirmed and hospitalized cases	1.6	1.3–2.0				
Confirmed and non-hospitalized cases	1.4	1.1–1.6				
Suspected cases	1.4	1.1–1.7				
Frequency of exiting the home during lockdown (ref: never or less than once a week)			0.001			0.001
Several times a day	0.6	0.4–0.9		**0.6**	0.4–0.9	
Once a day	0.7	0.5–0.9		**0.7**	0.5–0.9	
Several times a week	0.7	0.6–0.9		**0.7**	0.6–0.9	
Once a week	0.8	0.6–0.9		**0.7**	0.6–0.9	

	Bivariate regression analysis			Multivariate logistic regression analysis R^2^ = 0.19		
	OR	95% CI	*P* value	OR	95% CI	*P* value
Online teaching delivery (ref: none)			0.01			
Partial online teaching	1.1	0.9–1.3				
Total online teaching	0.9	0.7–1.0				
Time working at home (ref: no change)			< 0.0001			< 0.0001
Increased time working	1.1	0.9–1.4		1.0	0.8–1.3	
Reduced time working	1.9	1.6–2.2		**1.6**	1.3–1.9	
Postponement of final competition (Yes vs No)	1.4	1.1–1.7	0.0025			
Alcohol consumption (ref: none)			0.0392			
No change	0.9	0.7–1.1				
Increased consumption	1.3	1.0–1.6				
Reduced consumption	1.0	0.8–1.2				
Tobacco consumption (ref: none)			0.01			0.01
No change	1.3	0.8–2.0		1.3	0.8–2.0	
Increased consumption	1.6	1.2–2.1		**1.6**	1.2–2.1	
Reduced consumption	0.9	0.7–1.3		0.9	0.7–1.3	
Media entertainment (ref: not used) ¥			< 0.0001			0.0006
1-Ineffective	3.5	1.9–6.5		**2.5**	1.2–4.9	
Reading entertainment (ref: not used) ¥			< 0.0001			< 0.0001
1-Ineffective	1.9	1.4–2.6		**1.5**	1.04–2.1	
2	1.6	1.3–2.1		**1.7**	1.3–2.2	
Physical exercise (ref: not used) ¥			0.03			0.05
5-Very effective	0.6	0.5–0.7		**0.7**	0.5–0.9	
Snacking between meals (ref: not used) ¥			< 0.0001			0.04
5-Very effective	1.9	1.5–2.5		**1.4**	1.1–1.9	

Bolded numbers mean that the OR is statistically significant

OR, odds ratio: the probability of MCS < first quartile (i.e. impaired mental health); 95% CI = 95% confidence interval; OR < 1 decreased frequency of MCS < 1rst quartile; OR > 1 increased frequency of MCS < 1rst quartile

Probability of impaired mental health was increased for students without direct access to the outside with a garden, terrace or balcony (OR 1.8, 95% CI 1.4–2.2). Other risk factors included difficulties being able to isolate in the home (OR 1.4, 95% CI 1.2–1.7), noise inside the home (OR 1.5, 95% CI 1.2–1.8), noise outside the home (OR 1.3, 95% CI 1.1–1.6), and conflicts with occupants of the dwelling (OR 2.1, 95% CI 1.8–2.6) as well as someone in the home who had COVID-19 requiring hospitalization or not (OR 3.1, 95% CI 1.3–7.2; OR 1.6, 95% CI 1.1–2.3; respectively).

Impaired mental health was also associated with increased tobacco consumption (OR 1.6, 95% CI 1.2–2.1), self-perceived ineffectiveness of media entertainment (OR 2.5, 95% CI 1.2–4.9) or reading (OR 1.7, 95% CI 1.3–2.2) and self-perceived effectiveness of snacking (OR 1.4, 95% CI 1.1–1.9) to calm oneself. However, physical exercise perceived as effective for calming oneself was protective (OR 0.7, 95% CI 0.5–0.9).

Finally, students who went out several times a day, once a day, several times a week or once a week were at significantly less risk of impaired mental health than those who declared that they never went out during lockdown (OR 0.6, 95% CI 0.4–0.95; OR 0.7, 95% CI 0.5–0.9; OR 0.7, 95% CI 0.6–0.9 and OR 0.7, 95% CI 0.6–0;9, respectively).

## Discussion

The combined effects of the pandemic and the lockdown on the quality of life of students has not previously been assessed. Our study showed that an impaired mental health was associated with female sex, reduced learning time, reduced access to the outside, and other difficulties with the living situation. Other risk factors were tobacco consumption, ineffectiveness of media entertainment or reading, and effectiveness of snacking to calm oneself, while physical activity and accessing outside spaces were protective.

The COVID-19 pandemic has prompted most countries to opt for population containment and social distancing measures to control the spread of the virus. However, significant psychological effects have been reported in previous containment experiments [26]. This pandemic has already shown significant psychological symptoms related to anxiety, stress and depression [19, 27]. In addition, an association between psychological and physical symptoms has been shown [28]. Using a chain mediation model, the authors showed that the need for health information and the perceived impact of the pandemic were sequential mediators between physical symptoms resembling COVID-19 infection (predictor) and subsequent mental health status (outcome) [28].

The development of new guidelines to establish appropriate counseling, preventive and curative psychological actions online or for specific groups such as healthcare workers or older people have been identified as necessary measures in this situation [29]. Thus, the use of cognitive behavioral therapy (CBT), particularly Internet-based CBT, could aid in the prevention of the spread of infection during the pandemic [30–32]. Unfortunately, none of these measures currently address the particularly vulnerable student population.

Studies have demonstrated the importance of assessing the health-related quality of life of the student community [33, 34], a population group passing through an important phase of life. The epidemics SARS, Ebola, H1N1 and now COVID-19 have had a significant impact on the activity, behavior, morale and health of our fellow citizens. The first studies conducted in China on the impact of the current epidemic reported a significant amount of anxiety and depressive disorders as well as sleep disorders. Other studies have suggested a risk of increased suicidal behavior, psychotic symptoms, psychosomatic symptoms, symptoms of post-traumatic stress and consumption of psychoactive substances (alcohol, tobacco, etc.). The situation of the lockdown and its psychosocial and economic consequences but also fear oneself, and one's loved ones, of contamination, the virus, the illness and its consequences (serious somatic disorders and death) act on the mental health of students. Moreover, long periods of social isolation are well known to be associated with mental health problems, post-traumatic stress symptoms, avoidance behaviors and family conflicts [35]. In our study, mental health status of post-secondary students was closer to that of chronically ill young adults than the general population [36] and higher than that of Mexican students; the MCS was close to 23.7 with the presence of moderate depressive symptoms versus 39 with severe anxiety [37]. However, this comparison is cautioned because of the different culture and educational system between the two populations.

Another element that appears to have played a major role in the impact of the pandemic on the mental health of the population is the nature of the government's response, particularly the timeliness with which restrictive measures (lockdown) were put in place. A combined systematic review and meta-analysis study showed that a rapid response of restrictive measures by the government moderated the impact of COVID-19 on the mental health of the population. In light of the results of our study, this finding [38] may be more complex to interpret for the student population, as strict lockdown likely had a deleterious effect on their mental health, particularly for students who experienced confinement in student housing and those who chose to return to live with their parents during this period. These restrictive measures should have been accompanied, as soon as they were put in place, by measures to accompany and support students who are particularly exposed to the anxiety caused by this pandemic.

In the current study, among socio-demographic characteristics, only female sex was associated with increased likelihood of impaired perceived mental health. This result differed from a previous study finding no significant effect of sex on quality of life among undergraduate dentistry students in social isolation due to the COVID-19 pandemic [35] but agree with those of studies finding lower quality of life reported by female than male students or depressive symptoms more frequent in young women [38, 39]. This result is not surprising because if we look at the main dimensions of life that shape quality of life, women are more often disadvantaged overall than men, either in the general population or those with various chronic diseases [40–42].

As a direct consequence of the closure of universities, some students experienced a decrease in learning time, with a negative effect on their mental health. The reasons for this decrease could be inherent to the university system, some students having more difficulty than others in organizing distance learning. Previous work has effectively shown that performing distance education was significantly associated with good quality of life among students in isolation for COVID-19 [35]. This observation could also be due to factors intrinsic to students such as decreased motivation in this uncertain period. When students are not motivated, their level of engagement is reduced [43]. This phenomenon should be the subject of in-depth reflection by university officials to limit disparities between courses and ensure that students’ learning is properly monitored.

The type of housing and its effect on students’ mental health was a much-anticipated outcome of this study. Not having direct access to the outdoors with a garden, terrace or balcony had a significant impact on their mental health during the lockdown. This outcome was also predictable, but the study was able to quantify it. This finding is consistent with a study that found that an unfavorable home environment, including poor quality and non-functioning housing, lack of green space, noise and air pollution, was related to depressed mood [44]. Indeed, students living in university rooms may have had more mental health problems during the lockdown than those living in parental housing or another place with access to a private outdoor space. More surprisingly, the situation of some students who may have been confined to the family home also exposed them to risk. Indeed, difficulties isolating within the home, noise pollution in the home, proximity to an infected relative and conflicts with a family member were associated with increased likelihood of impaired mental health. The development of resilience may offer a feasible intervention and the benefits of such preparation will likely extend further, with strengthened resilience aiding in the transition from a student to an adult earning a living [45]. These observations support the need to develop interventions to support students who are isolated and potentially at risk. This support could include digital forms of study groups, peer group sessions and psychological interventions.

We observed that increased tobacco use was associated with impaired mental health. The factors underlying this association are still being determined, and given the cross-sectional design of our study, we caution the interpretation of this finding. Nevertheless, this result seems to be consistent with the literature showing that smokers are more anxious than non-smokers [46]. However, some students used psychoactive substances to help cope with stress during the pandemic. People relying on negative coping methods such as drinking and smoking and others using self-management relaxing hobbies including physical exercise or reading have been previously described [47].

This study has a number of limitations. First, the representativeness of the sample is limited, as it is a sample of voluntary participants, with an over-representation of women, which may have led to an under-evaluation of impaired mental health. Second, this cross-sectional study is useful in understanding the immediate or short-term impact apparent at a certain time point. However, the limitation of a cross-sectional design is that it cannot conclude on the long-term impact of COVID- 19, given that certain pre-existing vulnerabilities and high-risk factors could be multiple, ongoing or recurrent, and also the manner by which they work may vary. Consequently, there is a pressing need for longitudinal and developmental studies to be able to reveal the multiple layers of dynamic determinants playing a role during this time of global crisis [47, 48].

Third, the students were recruited from one of the French areas that was the most substantially affected by COVID-19, which limits the generalizability of these results to all students. Then, despite the large number of determinants included in the analyses, the multivariable model explained 19% of the explained variance; thus, other factors, such as anxiety due to media coverage and daily accurate information regarding the infection rates and number of deaths, were not accounted for in our study and should be included in future studies. Finally, the COVID-19 pandemic was found to cause hemodynamic changes in the brain [49]. This study used self-reported questionnaires to measure psychiatric symptoms and did not make a clinical diagnosis, the gold standard for establishing a psychiatric diagnosis being the structured clinical interview.

However, this study also has strengths. First, the large sample size (4018 respondents) allowed for a robust analysis and extracting solid tendencies and associations. Also, this is an early study that offers a unique opportunity to investigate the mental impact of the COVID-19 pandemic in a French student environment. Second, it provides valuable information about the current situation which may be useful gaining insights the need for a concerted action into the situation in other universities or in possible future global crises. Third, this study provides invaluable information on the self-perceived mental health of students in a French area particularly affected by COVID-19. Finally, our results bring attention to the findings that health initiatives for students should include improvements in learning and living environments. Simply developing resources to facilitate online guidance and lectures to offer strategies for managing anxiety and building a campus environment that offers access to a private outside space for students are essential, as these actions might have benefits for mental health.

Similar to a recent study that explored the perception and willingness of health care workers to be vaccinated against COVID-19 [50], it is essential that future research focus on students' perceptions of vaccination to better understand the barriers to vaccination and to develop education programs that incorporate the specificities of the student population.

## Conclusion

This study showed that the living conditions for post-secondary students during the COVID-19 lockdown in France had a clear impact on their mental health. This study highlights the need for a concerted action plan between university officials, teachers and students to meet the psychosocial and mental health needs of the students most at risk during a lockdown. There is a need to improve prevention and access to distance education as well as an urgent need to put in place measures to develop healthy coping strategies during the current crisis. Innovative mental health policies targeting students but more broadly all vulnerable population groups are needed, with direct and digital collaboration networks of psychiatrists and psychologists.

## Data Availability

The datasets used and analysed during the current study are available from the corresponding author on reasonable request.

## Abbreviations

COVID-19: Coronavirus disease 2019
WHO: World Health Organization
PIMS-COV19: Psychological Impact of the COVID-19 study
SF-12: Short-form 12 items (questionnaire)
MCS: Mental Component Score (of SF-12)

## References

[1] Zhu N, Zhang D, Wang W, Li X, Yang B, Song J (2020). China Novel Coronavirus Investigating and Research Team. A novel coronavirus from patients with pneumonia in China, 2019. N Engl J Med.

[2] Phelan AL, Katz R, Gostin LO (2020). The novel coronavirus originating in Wuhan, China: challenges for global health governance. JAMA.

[3] World Health Organization. 2019-nCoV outbreak is an emergency of international concern. 2020. http://www.euro.who.int/en/healthtopics/emergencies/pages/news/news/2020/01/2019ncov-outbreak-is-an-emergency-of-international-concern

[4] Kang SJ, Jung SI (2020). Age-related morbidity and mortality among patients with COVID-19. Infect Chemother.

[5] Sahu KK, Mishra AK, Lal A (2020). COVID-2019: update on epidemiology, disease spread and management. Monaldi Arch Chest Dis.

[6] Regehr C, Glancy D, Pitts A (2013). Interventions to reduce stress in university students: a review and meta-analysis. J Affect Disord.

[7] Wang C, Pan R, Wan X, Tan Y, Xu L, McIntyre RS (2020). A longitudinal study on the mental health of general population during the COVID-19 epidemic in China. Brain Behav Immun.

[8] Auerbach RP, Alonso J, Axinn WG, Cuijpers P, Ebert DD, Green JG (2016). Mental disorders among college students in the World Health Organization World Mental Health Surveys. Psychol Med.

[9] Zhai Y, Du X (2020). Addressing collegiate mental health amid COVID-19 pandemic. Psychiatry Res.

[10] Snedden TR, Scerpella J, Kliethermes SA, Norman RS, Blyholder L, Sanfilippo J (2019). Sport and physical activity level impacts health-related quality of life among collegiate students. Am J Health Promot.

[11] Ye Z, Yang X, Zeng C, Wang Y, Shen Z, Li X, Lin D (2020). Resilience, social support, and coping as mediators between COVID-19-related stressful experiences and acute stress disorder among college students in China. Appl Psychol Health Well Being.

[12] Mahmoud JS, Staten R, Hall LA, Lennie TA (2012). The relationship among young adult college students' depression, anxiety, stress, demographics, life satisfaction, and coping styles. Issues Ment Health Nurs.

[13] Odriozola-González P, Planchuelo-Gómez Á, Irurtia MJ, de Luis-García R (2020). Psychological effects of the COVID-19 outbreak and lockdown among students and workers of a Spanish university. Psychiatry Res.

[14] Araújo FJO, de Lima LSA, Cidade PIM, Nobre CB, Neto MLR (2020). Impact of Sars-Cov-2 and its reverberation in global higher education and mental health. Psychiatry Res.

[15] Zhou SJ, Zhang LG, Wang LL, Guo ZC, Wang JQ, Chen JC (2020). Prevalence and socio-demographic correlates of psychological health problems in Chinese adolescents during the outbreak of COVID-19. Eur Child Adolesc Psychiatry.

[16] Ge Y, Xin S, Luan D, Zou Z, Bai X, Liu M, Gao Q (2020). Independent and combined associations between screen time and physical activity and perceived stress among college students. Addict Behav.

[17] Liu S, Liu Y, Liu Y (2020). Somatic symptoms and concern regarding COVID-19 among Chinese college and primary school students: a cross-sectional survey. Psychiatry Res.

[18] Cao W, Fang Z, Hou G, Han M, Xu X, Dong J, Zheng J (2020). The psychological impact of the COVID-19 epidemic on college students in China. Psychiatry Res.

[19] Bourion-Bédès S, Tarquinio C, Batt M, Tarquinio P, Lebreuilly R, Sorsana C (2021). Psychological impact of the COVID-19 outbreak on students in a French region severely affected by the disease: results of the PIMS-CoV 19 study. Psychiatry Res.

[20] Zhang MWB, Lim RBC, Lee C, Ho RCM (2018). Prevalence of internet addiction in medical students: a meta-analysis. Acad Psychiatry.

[21] Gao L, Gan Y, Whittal A, Lippke S (2020). Problematic internet use and perceived quality of life: findings from a cross-sectional study investigating work-time and leisure-time internet use. Int J Environ Res Public Health.

[22] Kaparounaki CK, Patsali ME, Mousa DV, Papadopoulou EVK, Papadopoulou KKK, Fountoulakis KN (2020). University students' mental health amidst the COVID-19 quarantine in Greece. Psychiatry Res.

[23] Umberson D, Montez JK (2010). Social relationships and health: a flashpoint for health policy. J Health Soc Behav.

[24] Gandek B, Ware JE, Aaronson NK, Apolone G, Bjorner JB, Brazier JE (1998). Cross-validation of item selection and scoring for the SF-12 Health Survey in nine countries: results from the IQOLA Project. International Quality of Life Assessment. J Clin Epidemiol.

[25] Hair JF, Anderson RE, Tatham RL, Black WC (1995). Multivariate data analysis.

[26] Hawryluck L, Gold WL, Robinson S, Pogorski S, Galea S, Styra R (2004). SARS control and psychological effects of quarantine, Toronto, Canada. Emerg Infect Dis.

[27] Wang C, Pan R, Wan X, Tan Y, Xu L, Ho CS, Ho RC (2020). Immediate psychological responses and associated factors during the initial stage of the 2019 coronavirus disease (COVID-19) epidemic among the general population in China. Int J Environ Res Public Health.

[28] Wang C, Chudzicka-Czupała A, Tee ML, Núñez MIL, Tripp C, Fardin MA (2021). A chain mediation model on COVID-19 symptoms and mental health outcomes in Americans, Asians and Europeans. Sci Rep.

[29] Bao Y, Sun Y, Meng S, Shi J, Lu L (2020). 2019-nCoV epidemic: address mental health care to empower society. Lancet.

[30] Ho CS, Chee CY, Ho RC (2020). Mental health strategies to combat the psychological impact of coronavirus disease 2019 (COVID-19) beyond paranoia and panic. Ann Acad Med Singap.

[31] Zhang MW, Ho RC (2017). Moodle: the cost effective solution for internet cognitive behavioral therapy (I-CBT) interventions. Technol Health Care.

[32] Soh HL, Ho RC, Ho CS, Tam WW (2020). Efficacy of digital cognitive behavioural therapy for insomnia: a meta-analysis of randomised controlled trials. Sleep Med.

[33] Lins L, Carvalho FM, Menezes MS, Porto-Silva L, Damasceno H (2016). Health related quality of life of medical students in a Brazilian student loan programme. Perspect Med Educ.

[34] Dalton WT, Schetzina KE, Pfortmiller DT, Slawson DL, Frye WS (2011). Health behaviors and health-related quality of life among middle school children in southern Appalachia: data from the winning with wellness project. J Pediatr Psychol.

[35] Silva PGB, de Oliveira CAL, Borges MMF, Moreira DM, Alencar PNB, Avelar RL (2021). Distance learning during social seclusion by COVID-19: improving the quality of life of undergraduate dentistry students. Eur J Dent Educ.

[36] Barlogis V, Mahlaoui N, Auquier P, Pellier I, Fouyssac F, Vercasson C (2017). Physical health conditions and quality of life in adults with primary immunodeficiency diagnosed during childhood: a French Reference Center for PIDs (CEREDIH) study. J Allergy Clin Immunol.

[37] Núñez-Rocha GM, López-Botello CK, Salinas-Martínez AM, Arroyo-Acevedo HV, Martínez-Villarreal RT, Ávila-Ortiz MN (2020). Lifestyle, quality of life, and health promotion needs in Mexican university students: important differences by sex and academic discipline. Int J Environ Res Public Health.

[38] Lee Y, Lui LMW, Chen-Li D, Liao Y, Mansur RB, Brietzke E (2021). Government response moderates the mental health impact of COVID-19: a systematic review and meta-analysis of depression outcomes across countries. J Affect Disord.

[39] Lim GY, Tam WW, Lu Y, Ho CS, Zhang MW, Ho RC (2018). Prevalence of depression in the community from 30 countries between 1994 and 2014. Sci Rep.

[40] Aslan H, Pekince H (2020). Nursing students' views on the COVID-19 pandemic and their percieved stress levels. Perspect Psychiatr Care.

[41] Baumann C, Erpelding ML, Perret-Guillaume C, Gautier A, Régat S, Collin JF, Guillemin F, Briançon S (2011). Health-related quality of life in French adolescents and adults: norms for the DUKE Health Profile. BMC Public Health.

[42] Kiely KM, Brady B, Byles J (2019). Gender, mental health and ageing. Maturitas.

[43] Aguilera-Hermida AP (2020). College students’ use and acceptance of emergency online learning due to COVID-19. Int J Educ Res Open.

[44] Rautio N, Filatova S, Lehtiniemi H, Miettunen J (2018). Living environment and its relationship to depressive mood: a systematic review. Int J Soc Psychiatry.

[45] O'Byrne L, Gavin B, McNicholas F (2020). Medical students and COVID-19: the need for pandemic preparedness. J Med Ethics.

[46] Ho CSH, Tan ELY, Ho RCM, Chiu MYL (2019). Relationship of anxiety and depression with respiratory symptoms: comparison between depressed and non-depressed smokers in Singapore. Int J Environ Res Public Health.

[47] Ye Z, Yang X, Zeng C, Wang Y, Shen Z, Li X, Lin D (2020). Resilience, social support, and coping as mediator between COVID-19-related stressful experiences and acute stress disorder among college students in China. Appl Psychol Health Well Being.

[48] Holmes EA, O'Connor RC, Perry VH, Tracey I, Wessely S, Arseneault L (2020). Multidisciplinary research priorities for the COVID-19 pandemic: a call for action for mental health science. Lancet Psychiatry.

[49] Olszewska-Guizzo A, Mukoyama A, Naganawa S, Dan I, Husain SF, Ho CS, Ho R (2021). Hemodynamic response to three types of urban spaces before and after lockdown during the COVID-19 pandemic. Int J Environ Res Public Health.

[50] Chew NWS, Cheong C, Kong G, Phua K, Ngiam JN, Tan BYQ (2021). An Asia-Pacific study on healthcare workers' perceptions of, and willingness to receive, the COVID-19 vaccination. Int J Infect Dis.

